# Some Diseases of the Male Genital System

**Published:** 1908-03-21

**Authors:** 


### Points in Surgery.

### SOME DISEASES OF THE MALE GENITAL SYSTEM.

### XI.—SYPHILITIC ORCHITIS.

The pathological changes produced by syphilis in the testis are essentially similar to those which it produces in other glandular organs. Syphilis is one of the diseases whose lesions are known as infective granulomata, wherein, as the result of a definite infection with a known micro-organism, a connective-tissue reaction is set up. The essential phenomenon is an overgrowth of small round nucleated connective tissue cells, which cannot be distinguished from the fixed connective tissue cells of the part except by their arrangement in masses. Whether these new-formed connective tissue cells are actually derived from the fixed connective tissue cells of the part or whether they are formed from the endothelial cells of the small arteries in the neighbourhood is a question over which there is some difference of opinion among eminent pathologists, and the matter is still *sub judice*. Now, the connective-tissue cell which proliferates in response to the stimulus of infection is the precursor of the fibroblast, and the final function of the fibroblast is to form fibrous tissue. In some cases, all the intermediate forms between round connective-tissue cell and adult fibrous tissue can be demonstrated in the same specimen: first the small round nucleated cell, then the spindle-shaped fibroblast, and finally the elongated fully-formed fibrous tissue-cell. If an entire organ is invaded by these connective tissue-cells, the ultimate result is a generalised fibrosis, which ends in great contraction (for the function of fibrous tissue is to contract) and consequent atrophy of any glandular structure owing to mechanical pressure. The organ is literally squeezed to death. But if the original focus of proliferation is localised and only one part of an organ is attacked, what is the result? The cells at the periphery of the part attacked attain their adult condition earlier than do the central ones, and sooner or later the focus consists of a number of small round cells walled in by a tough fibrous-tissue capsule. After a time the central cells die and caseate: this is the outcome of one or more of the following factors—the avascularity of the part owing to the toughness of its capsule and to the advanced endarteritis of the surrounding vessels, which is so characteristic a feature of syphilis. Perhaps, also, the actual toxin secreted by the organism may play a part. The result is a gumma which breaks down in the centre and forms a gummatous abscess. The pus which it contains is not true pus in the correct sense of the word, but consists of disintegrated connective-tissue cells. There is thus a striking analogy between syphilis and tuberculosis. Both are infective granulomata, both cause primarily a connective-tissue reaction, and both are associated, though this is not generally known, with endarteritis. The writer has seen sections of a cerebral artery from a case of definite cerebral tuberculosis, in which the endothelial coat was so greatly proliferated as to occlude completely the lumen of the vessel. Further than this, now that the micro-organism which causes syphilis has been definitely isolated, bacteriologists have found that it resembles the tubercle bacillus in many of its characteristics.

If we apply this description of the general pathological changes produced by syphilis to the testicle, we shall find no difficulty in making it fit the clinical phenomena. Thus we find a diffuse interstitial orchitis, in which the organ is enlarged. In the early stages it may be tender, but later testicular sensation is lost. This is due to the fact that the whole glandular structure has been replaced by other elements. If the progress of such a case be watched, the most common termination is a gradual diminution in size, with a sense of increasing hardness on palpation. In this process the connective-tissue cells are being converted into fibrous tissue. And, finally, the whole organ may go on shrinking until it is no larger than an ordinary bean and consists of nothing more or less than a mass of fibrous tissue. If this is bilateral, the patient is, of course, absolutely sterile.

In other cases the enlargement is even from the first a localised affair, and in these, as one might suppose, testicular sensation is not completely lost. It is a mistake to suppose that testicular sensation is invariably absent in syphilitic orchitis; if this is accepted as an infallible means of diagnosis many errors will be made. It is this form of the disease which goes on to gumma formation and to the so-called gummatous abscess. Eventually the caseous material makes its way to the surface through the weakest part of its fibrous-tissue capsule and becomes superficial by involving the overlying scrotum. When this has occurred it is more than likely that sepsis is superadded owing to the introduction of pyogenic micro-organisms, and the resulting condition is somewhat intractable. For, although the syphilitic orchitis will often improve remarkably under treatment, a hernia testis is left which will demand surgical interference. The adnexa of the testis, the epididymis, and the vas deferens generally escape in syphilis, but the tunica vaginalis suffers, as is evidenced by the frequent association of a hydrocele with a syphilitic orchitis.

The treatment consists primarily in the general treatment of the disease. The administration of potassium iodide results in remarkable improvement in the local condition, because it helps in the absorption of connective tissue; but it should be remembered that it does not cure the disease. Mercury should, therefore, always be given with it, because a man with a syphilitic testicle must be regarded as having evidence of more or less active syphilis: and, more than this, the mercury should be continued for at least a month or two after all the local symptoms have disappeared. Not a few cases have been known to recur for want of this precaution.

